# Optimization of parallel coiled cavities of different depths in microperforated panel sound absorbers

**DOI:** 10.1038/s41598-025-85171-3

**Published:** 2025-01-09

**Authors:** Hequn Min, Huading Lou, Yuchen Zhao

**Affiliations:** Key Laboratory of Urban and Architectural Heritage Conservation, Ministry of Education, School of Architecture, Southeast University, 2# Sipailou, Nanjing, 210096 China

**Keywords:** Microperforated panel, Sound absorber, Broadband sound absorption, Coiled-up cavity, Civil engineering, Acoustics

## Abstract

This paper presents a microperforated panel (MPP) sound absorber with parallel coiled-up-cavities of different-depths (PCD) and the corresponding optimization on their cavities. In this study, an analytical model is initially proposed for estimating the cavity depths of the PCD-MPP absorber upon normal incidence absorption coefficient evaluation at given resonance frequencies. Cavity effective depths and normal incidence absorption coefficient are evaluated after coiling up cavities for a compact structure. Numerical simulations with the finite element method and experiments are conducted for validations. Subsequently, a design process is suggested on the basis of the proposed model for sound absorption optimization. Results show that, absorption coefficient from the proposed analytical model agrees well with finite element simulations and experiments. It is also shown that, for the effective depth evaluation of the coiled-up cavities of PCD-MPP absorbers, the diagonal lines of subchannels of coiled-up cavities with a coiled-up angle of 180° can accurately represent the effective depths, while the combination of centerlines of subchannels and quarter arc inside the coiled-up area is more suitable for those with a coiled-up angle of 90°. Optimization investigation shows that, PCD-MPP absorbers can have high absorption performance with the averaged and maximum absorption coefficient of 0.91 and 0.98 within the target bandwidth of 400–1600 Hz, where the absorber thickness can stay below 65 mm. This work can provide valuable guidelines for the design of sound absorbers for broadband absorption.

## Introduction

Noise is considered as one of the annoying pollution sources that have a negative impact on the public^1–5^, and spectra of different noise types, such as ventilation and air conditioning system noise, irrelevant speech, building equipment noise and human activity noise, differ from each other. For example, noise peaks of mechanical ventilation systems are usually located at low frequencies. With the increase of air flow speed, in-duct sound power level gradually increases and noise of broadband up to 2000 Hz or even 3000 Hz is generated^6^. Some types of noise emitted by building equipment, such as pumps and air blowers, may be only located at narrow bands. To deal with noise issues, a microperforated panel (MPP) absorber is a promising alternative to conventional porous absorber and is widely applied in noise control engineering.

Absorption performance of an MPP absorber can be improved by perforated parameter optimization^7–9^, compound absorber^10^ or multi-layer panels^11^. For example, increasing the number of perforated panels enhances better absorption performance over broadband frequency ranges, but increases the thickness of the absorber. Adjusting the forms of cavity division^12^ or using multi honeycomb-shaped sub-cavities^13,14^ can also broaden absorption bandwidth and achieve more resonance absorption peaks. A single layer MPP backed by parallel-arranged cavities of different depths also show high performance with wideband absorption, which has a thinner thickness than multiple layer serial-arranged MPPs. Comparing with the single layer MPP absorber, more absorption peaks are achieved when multi cavities with different depths are introduced^15,16^. To create more parallel-arranged cavities of different depths, Wang^17^ proposed a single layer MPP absorber backed with three parallel-arranged cavities of different depths after the study on acoustic properties of the MPP absorber backed by a trapezoidal cavity^18^. Later, Wang et al.^19^ presented a basic module of an MPP absorber array consisting of four parallel-arranged MPP absorbers with different cavity depths, and the whole array was created by arranging the basic modules in a periodically repeating pattern. In addition, absorption performance can be further improved by using the serial-parallel-coupled MPP absorbers^20^ or acoustic metamaterial^21,22^.

A hybrid metamaterial absorber constructed with an MPP and coiled-up channels shows broadband low-frequency absorption with deep-subwavelength thickness, and the frequency of the absorption peak could be tuned by adjusting the geometry parameters of the MPP and the channel folding numbers^23,24^. A coiled-up channel strategy is used to design compact back cavities for low-frequency absorption, tremendously decreasing the space occupation of conventional Helmholtz resonators and MPP absorbers. Sound absorbers with coiled-up cavities, therefore, are widely investigated. For instance, Li and Assouar^25^ presented a metasurface-based absorber consisting of a perforated plate and a coiled coplanar air chamber, which was capable of achieving the total absorption of acoustic wave in an extremely low frequency region in a deep subwavelength thickness. Wang et al.^26^ presented a deep-subwavelength absorber based on the coiled-up space. By adjusting a partition panel in the cavity to form an unequal-section channel, it was found that the resonance frequency of the absorber was easily tuned and near-total absorption was acquired under a fixed deep-subwavelength thickness. Almeida et al.^27^ proposed an acoustic metamaterial based on an MPP coupled to a multi-cavity of coiled-up spaces, and it was demonstrated that the proposed absorber presented a scale of deep-subwavelength since its total thickness was 0.033*λ*. Wang et al.^28^ and Cheng et al.^29^ also investigated the absorption performance of the acoustic metamaterial based on the coiled-up cavities. Absorbers backed with arch-like channels are squeezed within a compact disk-shaped volume with a small thickness of deep wavelength for low absorption in limited-volume circumstances. Compared with a uniform cross-section, varied channel widths can effectively lower the operating frequency^30,31^. Besides, an acoustic metamaterial, consisting of porous material lining to a coiled-up air channel, combines the acoustic advantages of coiled-up space and porous material to achieve efficient sound absorption at low frequencies. The metamaterial with high folding number shows deep subwavelength absorption, while it shows good broadband sound absorption with low folding number^32^.

It is often the case in noise control engineering that the target bandwidth with high absorption performance of an absorber should cover the main bandwidth of noise peaks. Parallel coiled-up-cavities of different-depths (PCD) are thus utilized for obtaining multiple resonances and broadband absorption performance to cover the target bandwidth. However, how the resonance frequency of the PCD-MPP absorber is influenced by the cavity depth is unknown, and the appropriate number of cavity for efficient absorption has not been discussed in previous works. Besides, it is interesting to investigate methods of cavity coiling up for a compact structure, and to evaluate the absorption performance after coiling up cavities.

Therefore, the aim of the current work is to propose a systematic design method on the cavity of a PCD-MPP absorber with coiled-up cavities, attempting to provide useful guidelines in noise control engineering. Specifically, the analytical model of absorption coefficient of the PCD-MPP absorber with straight cavities is first reviewed. An analytical model is then proposed to estimate the depth and number of cavities upon a given target bandwidth, and the accuracy is also evaluated. Besides, an analytical model based on the impedance transfer method is presented to evaluate absorption coefficient after cavity coiling up, where the effective depth estimation of the coiled-up cavity is also discussed. Experiments are finally performed to measure the normal incidence absorption coefficient of a PCD-MPP absorber with wideband absorption in an impedance tube.

## Geometry

**Fig. 1:**
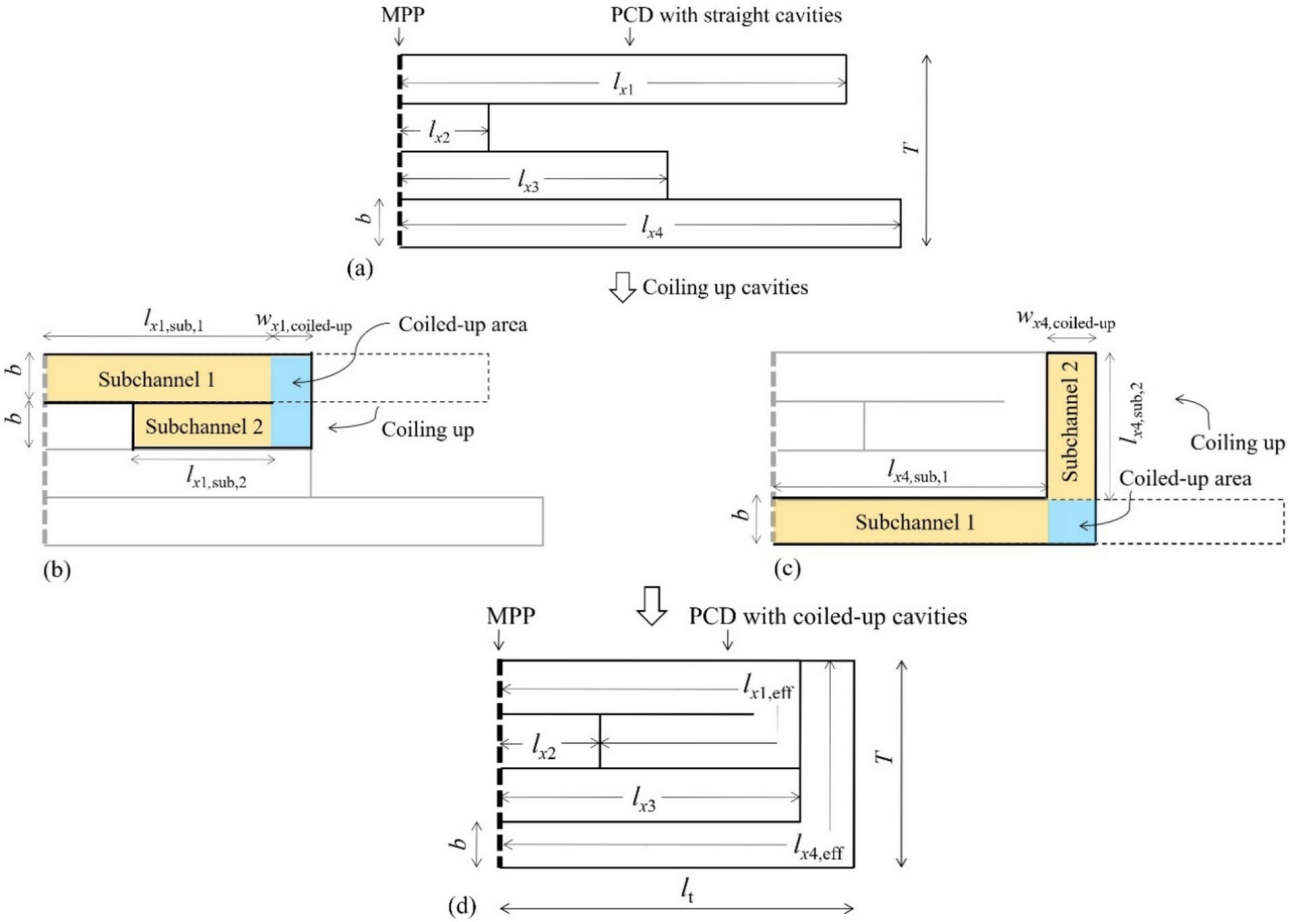
(**a**) a PCD-MPP absorber with four straight cavities of *l*_*x*1_, *l*_*x*2_, *l*_*x*3_, *l*_*x*4_ in depth; (**b**) and (**c**) coiling up the cavities, *l*_*x*1_ and *l*_*x*2_ in depth, with the coiled-up angle of 180° and 90°, respectively, where the subchannels and coiled-up area of each coiled-up cavity are marked with yellow and blue; (**d**) a PCD-MPP absorber with coiled-up cavities. The cavity width, *b*, ignores the thickness of walls between cavities. Parameters of the MPP include the perforation rate, *σ*, perforated diameter, *d*, and panel thickness, *t*.

A PCD-MPP absorber is composed of a surface MPP backed by an array of PCD. An example with four straight cavities is shown in Fig. 1a. *b*, *T* = *N* × *b*, respectively, represent the width of each cavity (ignoring the thickness of walls between cavities) and the total width of the absorber, where *N* is the number of cavities. The lengths of each straight cavity are *l*_*x*1_, *l*_*x*2_, *l*_*x*3_, *l*_*x*4_, respectively, and the cavity depth sequence is defined as [*l*_*x*1_, *l*_*x*2_, *l*_*x*3_, *l*_*x*4_]. Parameters of the MPP include the perforation rate, *σ*, perforated diameter, *d*, and panel thickness, *t*. To obtain an absorber with a rectangular cross section, straight cavities, *l*_*x*1_ and *l*_*x*4_ in depth in Fig. 1a, are coiled up with a coiled-up angle of 180° and 90°, respectively, which is shown in Fig. 1b and c. The coiled-up area of each coiled-up cavity is marked with blue, and subchannels are illustrated with yellow, where the one close to the surface MPP is defined as the first subchannel. A PCD-MPP absorber with coiled-up cavities in Fig. 1d, whose thickness is *l*_t_, is finally obtained, and the effective depths (the effective sound propagation paths) of the two coiled-up cavities are *l*_*x*1,eff_ and *l*_*x*4,eff_, respectively.

## Theory

### Analytical model of the PCD-MPP absorber with straight cavities

The classical analytical model of the PCD-MPP absorber with straight cavities is reviewed in this section. The specific characteristic impedance of an MPP^33^ is defined as

1 $${Z}_{\text{MPP}}=r+j\omega m ,$$

where *ω* = 2π*f* is the angular frequency. *r* and *m* represent acoustic resistance and mass reactance, respectively, and can be calculated as

2 $$r=\frac{32\eta t}{\sigma {\rho }_{0}{c}_{0}{d}^{2}}\left[{\left(1+\frac{{k}^{2}}{32}\right)}^\frac{1}{2}+\frac{\sqrt{2}}{32}k\frac{d}{t}\right] ,$$

3 $$\omega m=\frac{\omega t}{\sigma {c}_{0}}\left[{\left(1+\frac{{k}^{2}}{2}\right)}^{-\frac{1}{2}}+0.85\frac{d}{t}\right] ,$$

where *η*, *ρ*_0_, *c*_0_ represent air viscosity coefficient, air density and sound velocity, respectively. *σ*, *d*, *t* denote perforated rate, perforated diameter, and panel thickness of MPP, respectively. Perforated constant *k* is defined as *d* $\sqrt{f/10}$, where *f* represents sound frequency in Hz. According to Maa’s theory^33^, the specific characteristic impedance *Z*_*c*_ of a straight cavity with equal cross section behind the MPP with a depth of *l*_*x*_ is

4 $${Z}_{c}=-j\text{cot}({\omega l}_{x}/{c}_{0}) .$$

After obtaining the specific characteristic impedance of the MPP and cavity, its surface admittance function *G*(*x*) is introduced:

5 $$G\left(x\right)=\frac{1}{{Z}_{MPP}+{Z}_{c}} .$$

Equation (6) can be obtained according to the relationship between particle velocity on the surface of the absorber and sound pressure, that is, *ρcv*_*z*_(*x*,0) = -*G*(*x*)*p*(*x*,0).

6 $$\text{cos}{\theta }_{e}{P}_{e}-\sum_{n=-\infty }^{+\infty }\frac{{\gamma }_{n}}{{k}_{0}}{A}_{n}{e}^{-\frac{jxn2\pi }{T}}=-G\left(x\right)\left[{P}_{e}+\sum_{n=-\infty }^{+\infty }{A}_{n}{e}^{-jxn2\pi /T}\right] .$$

*θ*_*e*_, *P*_*e*_, *A*_*n*_ represent, respectively, sound incidence angle, unit sound pressure, and amplitude coefficient. *γ*_*n*_ is the number of spatial harmonic waves in the axial direction of the tube and can be expressed as

7 $${\gamma }_{n}=-j{k}_{0}\sqrt{{\left(\text{sin}{\theta }_{e}+n\frac{\lambda }{T}\right)}^{2}-1} .$$

Radiation harmonic exponent *n*_*s*_ should be controlled according to Eq. (8) to ensure that sound wave can radiate to far field.

8 $${\left({\text{sin}\theta }_{e}+{n}_{s}\frac{\lambda }{T}\right)}^{2}\le 1 .$$

Fourier transform of period *T* is then conducted as

9 $$G\left(x\right)=\sum_{n=-\infty }^{+\infty }{g}_{n}{e}^{-jxn2\pi /T},$$

10 $${g}_{n}=\frac{1}{T}{\int }_{0}^{T}G\left(x\right){e}^{jxn2\pi /T}{d}_{x} .$$

Equation (9) then substitutes into Eq. (6). Equation (6) is multiplied by *e*^j*xm*2π/*T*^ at the both sides of the equation, following integral operation from 0 to *T*.

11 $$\sum_{n=-\infty }^{+\infty }{A}_{n}\left({g}_{m-n}+{\delta }_{m,n}\frac{{\gamma }_{n}}{{k}_{0}}\right)={P}_{e}\left({\delta }_{m,0}\text{cos}{\theta }_{e}-{g}_{m}\right) ,$$

where

12 $${\delta }_{m,n}=\left\{\begin{array}{c}1 m=n\\ 0 m\ne n\end{array} ,\text{m},\text{ n}\in (-\infty ,+\infty )\right..$$

A (4*N* + 1) × (4*N* + 1) matrix can be obtained by limiting the value range of m and n to [− 2*n*, 2*N*], and the amplitude coefficient *A*_*n*_ can be calculated by solving the matrix. Finally, oblique incidence sound absorption coefficient of a PCD-MPP absorber can be expressed as Eq. (13)^16^.

13 $$\alpha \left({\theta }_{e}\right)=1-{\left|\frac{{A}_{0}}{{P}_{e}}\right|}^{2}-\frac{1}{\text{cos}{\theta }_{e}}\sum_{{n}_{s}\ne 0}{\left|\frac{{A}_{{n}_{s}}}{{P}_{e}}\right|}^{2}\sqrt{1-{\left(\text{sin}{\theta }_{e}+{n}_{s}\lambda /T\right)}^{2}} .$$

And the normal incidence absorption coefficient of the PCD-MPP absorber,$\alpha ({\theta }_{0})$, is given by

14 $$\alpha \left({\theta }_{0}\right)=1-{\left|\frac{{A}_{0}}{{P}_{e}}\right|}^{2}-\sum_{{n}_{s}\ne 0}{\left|\frac{{A}_{{n}_{s}}}{{P}_{e}}\right|}^{2}\sqrt{1-{\left({n}_{S}\lambda /T\right)}^{2}} .$$

The accuracy of this analytical model of the PCD-MPP absorber with straight cavities has been validated in our previous work^21^ by comparing with the sound absorption coefficient by analytical model, numerical simulation and experiments.

### Prediction on the depth and number of the cavity

The appropriate number and interval of the design values of resonance frequency,* f*_*α,max,des*_, are of significance in the design process, so as to cover the target bandwidth of the given noise. Cavity depth and cavity number of the PCD-MPP absorber in this work is determined according to Maa’s theory^33^. Specifically, the lower frequency of the target bandwidth is determined as the first *f*_*α,max,des*_. To determine the second *f*_*α,max,des*_, the half-absorption bandwith, *B*, is introduced to describe the interval between the two adjacent resonance frequencies. It is defined as the frequency interval where the absorption coefficient is one-half of the maximum value, and the maximum value is located at the resonance frequency. The absorption coefficient is one-half of the maximum value at the lower frequency and upper frequency given by

15 $$\omega m-\text{cot}\left(\frac{\omega {l}_{x}}{{\text{c}}_{0}}\right)=\mp \left(1+r\right),$$

where the upper frequency of the half-absorption bandwith of the first* f*_*α,max,des*_ is defined as the second *f*_*α,max,des*_ of the PCD-MPP absorber. Repeat this process by using Eq.  (15) until the interval between the fisrt and last *f*_*α,max,des*_ covers the target bandwidth.

After determining the values of *f*_*α,max,des*_, the analytical model of a single layer MPP absorber proposed by Maa^33^ is then introduced to estimate cavity depths, upon the given *f*_*α,max,des*_ and MPP parameters, which is given by

16 $$\omega m-\text{cot}\frac{\omega {l}_{x}}{{c}_{0}}=0 .$$

### Analytical model of the PCD-MPP absorber with coiled-up cavities

Straight cavities are coiled up with a coiled-up angle of 180° or 90° to obtain a PCD-MPP absorber with coiled-up cavities. This subsection is to discuss how to define the effective depth of a coiled-up cavity, so as to make an accurate evaluation on the absorption coefficient. Actually, there are variable methods to estimate the effective depth of a coiled-up cavity from published works, such as using the total length of centerlines along the sound propagation in subchannels^34,35^, using the total length of diagonal lines of subchannels^36–39^, using the combination of centerlines of subchannels and quarter arc inside the coiled-up area^40^, but which method is suitable for evaluating the effective depth of a coiled-up cavity in this work is unknown.

**Fig. 2:**
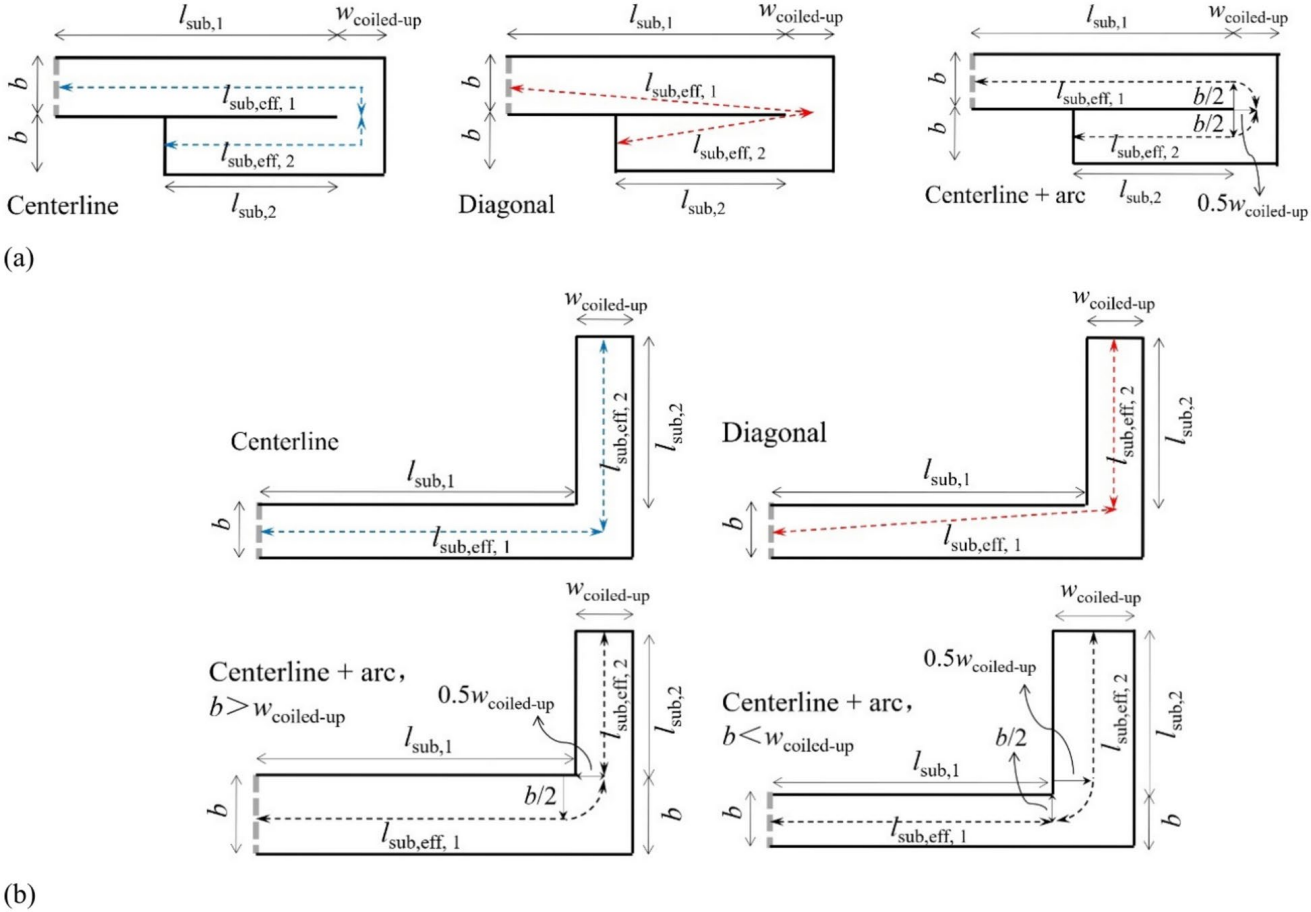
Schematics of methods for estimating the effective depth of a coiled-up cavity with a coiled-up angle of (**a**) 180° or (**b**) 90°, where the dotted line with arrows represents the effective length of each subchannel.

Figure 2 illustrates the three methods, where the dotted line with arrows represents the effective length of each subchannel. For a coiled-up cavity with a coiled-up angle of 180°, as shown in Fig. 2a, the effective length of each subchannel, *l*_sub,eff,*i*_, is expressed by

17 $$l_{{{\text{sub}},{\text{eff}},i}} = l_{{{\text{sub}},{\text{i}}}} + \frac{1}{2}w_{{{\text{coiled}} - {\text{up}}}} + \frac{1}{2}b,\,{\text{Centerline}}$$

18 $$l_{{{\text{sub}},{\text{eff}},i}} = \sqrt {\frac{1}{4}b^{2} + \left( {l_{{{\text{sub}},i}} + \frac{1}{2}w_{{{\text{coiled}} - {\text{up}}}} } \right)^{2} } ,{\text{ Diagonal}}$$

19 $$l_{{{\text{sub}},{\text{eff}},i}} = l_{{{\text{sub}},i}} - \frac{1}{2}b + \frac{1}{2}w_{{{\text{coiled}} - {\text{up}}}} + \frac{1}{4}\pi b,{\text{ Centerline }} + {\text{ arc}}$$

When the coiled-up angle is 90°, as shown in Fig. 2b, the effective length of each subchannel, *l*_sub,eff,*i*_, is expressed by

20 $$l_{{{\text{sub}},{\text{eff}},i}} = l_{{{\text{sub}},1}} + \frac{1}{2}w_{{{\text{coiled}} - {\text{up}}}} { },{ }l_{{{\text{sub}},2}} + \frac{1}{2}{\text{b}},{\text{ Centerline}}$$

21 $$l_{{{\text{sub}},{\text{eff}},i}} = \sqrt {\frac{1}{4}b^{2} + \left( {l_{{{\text{sub}},1}} + \frac{1}{2}w_{{{\text{coiled}} - {\text{up}}}} } \right)^{2} } ,{ }l_{{{\text{sub}},2}} ,{\text{ Diagonal}}$$

22 $$l_{{{\text{sub}},{\text{eff}},i}} = \left\{ {\begin{array}{*{20}l} {l_{{{\text{sub}},1}} - \frac{1}{2}b + \frac{1}{2}w_{{{\text{coiled}} - {\text{up}}}} + \frac{1}{4}\pi b, l_{{{\text{sub}},2}} ,} & {b > w_{{{\text{coiled}} - {\text{up}}}} ,} & {} \\ {} & {} & {{\text{Centerline }} + {\text{ arc}}} \\ {l_{{{\text{sub}},1}} ,{ }l_{{{\text{sub}},2}} + \frac{1}{2}b - \frac{1}{2}w_{{{\text{coiled}} - {\text{up}}}} + \frac{1}{4}\pi w_{{{\text{coiled}} - {\text{up}}}} ,} & {b < w_{{{\text{coiled}} - {\text{up}}}} ,} & {} \\ \end{array} } \right.$$

From Eq. (17) to Eq. (22), *l*_sub,*i*_ denotes the length of the *i*-th subchannel of a coiled-up cavity, *i* = 1, 2. *w*_coiled-up_ represents the width of the coiled-up area.

The analytical model in subsection "Analytical model of the PCD-MPP absorber with straight cavities", which is based on Maa’s theory, does not consider the impedance change caused by the bend of the coiled-up cavity. An analytical model is thus proposed in this work. Generally, a coiled-up cavity can be modeled as an array of equivalent coaxial straight multiple subchannels, whose surface impedance, *Z*_1_, at the cavity top entrance can be obtained by impedance transfer method. For each subchannel of a coiled-up cavity, the surface impedance, *Z*_*i*_, at the top entrance can be express as

23 $${Z}_{i}=\frac{\left(\frac{{\text{Z}}_{\text{i}+1}}{{\upxi }_{\text{i}+1}}\right)\text{cot}\left(\frac{\omega {l}_{\text{sub},\text{eff},i}}{{c}_{0}}\right)+j}{\text{cot}\left(\frac{\omega {l}_{\text{sub},\text{eff},i}}{{c}_{0}}\right)+j\left(\frac{{Z}_{i+1}}{{\xi }_{i+1}}\right)} , i=1, 2, \cdots , n-1,$$

24 $${Z}_{i}=-j\text{cot}\left(\frac{\omega {l}_{\text{sub},\text{eff},i}}{{c}_{0}}\right) , i=n,$$

where *i* represents the *i*-th subchannel, and *n* denotes the number of subchannels of a coiled-up cavity. *ξ*_*i*+1_ is the ratio *A*_*i*+1_ : *A*_*i*_, where *A*_*i*+1_, *A*_*i*_ are the cross-sectional areas of the (*i* + 1)-th and *i*-th subchannels, respectively.

After obtaining the surface impedance, *Z*_1_, at the top entrance of a coiled-up cavity, the oblique incidence sound absorption coefficient of a PCD-MPP absorber with coiled-up cavities can be calculated by using Eq. (5) to Eq. (13).

## Numerical results and discussions

In this paper, based on the standard ISO 10,534–2^41^, numerical simulations with the finite element method (FEM) are conducted by a commercial FEM package, COMSOL Multiphysics 6.0, to evaluate the normal incidence absorption coefficient and sound field of the PCD-MPP absorber. The FEM model consists of an impedance tube and a tested PCD-MPP absorber mounted at the end of the tube, and they are of equal dimension in cross section. The plane wave is incident from the left side of the tube and reflected on the surface of the MPP. The MPP is considered as an interior impedance boundary, and the specific characteristic impedance is defined according to Eq. (1). The acoustics-vibration coupling effect is not considered in this paper, and the interior partition walls of the PCD, exterior walls of the impedance tube and absorber are set as acoustically rigid boundaries with the ignorance of thickness.

### Accuracy evaluation of the model for estimating the cavity depths of the PCD-MPP absorber

The interactions between the local resonance and the harmonic of cavities in different depths may cause the resonance frequency shift of a certain cavity^21^, leading to the differences of resonance frequencies between the design and actual values. Therefore, the accuracy of Eq. (16) for estimating cavity depths of the PCD-MPP absorber should be evaluated. Specifically, values of *f*_*α,max,des*_ are first assumed, and the corresponding cavity depths of the PCD-MPP absorber are calculated by using Eq. (16) upon the given perforation parameters. The actual values of resonance frequencies,* f*_*α,max,act*_, of each cavity can be determined after obtaining the absorption coefficient of the PCD-MPP absorber, and then the accuracy of Eq. (16) is evaluated by comparing the resonance frequencies between the design and actual values.

**Fig. 3:**
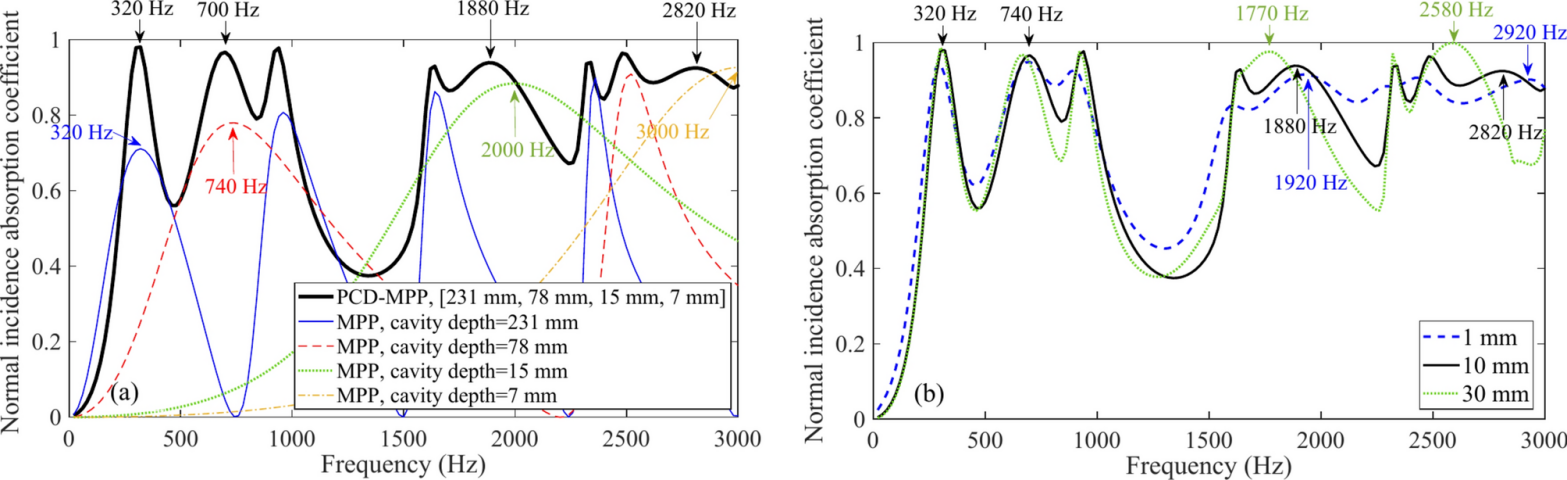
(**a**) Comparisons of resonance frequencies of a PCD-MPP absorber between the design and actual values (MPP parameters: *d* = *t* = 0.4 mm, *σ* = 1.8%; cavity width = 10 mm); (**b**) effects of cavity widths on the actual values of resonance frequencies (MPP parameters: *d* = *t* = 0.4 mm, *σ* = 1.8%; cavity depth sequence = [231 mm, 78 mm, 15 mm, 7 mm]).

Four design values of *f*_*α,max,des*_, 320 Hz, 740 Hz, 2000 Hz and 3000 Hz, are first listed, and the corresponding cavity depths, 231 mm, 78 mm, 15 mm and 7 mm, are obtained by using Eq. (16) upon the given perforation parameters of [*d* = *t* = 0.4 mm, *σ* = 1.8%] and cavity width of 10 mm. Then the normal incidence absorption coefficient of the PCD-MPP absorber is calculated, as shown in Fig. 3a. It should be mentioned that the actual values of *f*_*α,max,act*_ of the absorber is determined by referring the corresponding single layer MPP absorber with the same perforation parameters, and the values of* f*_*α,max,act*_ are marked in Fig. 3a. Small differences between *f*_*α,max,des*_ and *f*_*α,max,act*_ are observed at frequency 740 Hz, 2000 Hz and 3000 Hz with the errors of 5.4%, 6.0% and 6.0%. Furthermore, effects of cavity widths (1 mm, 10 mm and 30 mm) on the accuracy of Eq. (16) are also investigated in Fig. 3b, and the actual resonance frequencies are marked. It can be seen that, differences between the design and actual values turn larger with the increase of frequency or cavity width, with an error from 0 to 14.0%.

In Fig. 3, narrow bandwith and weak interactions between absorption peaks can be found at low frequencies, and well agreement between the design and actual values is thus achieved. However, when the design values are located at higher frequencies, such as at 2000 Hz, the coupling effect of the local resonance of the 15 mm-depth cavity and the harmonic of the 231 mm-depth cavity leads to the shift of actual resonance frequency at around 1880 Hz (see Fig. 3a), and the error between the design and actual value thus turns larger. Although the accuracy of the model for predicting the cavity depth of the PCD-MPP absorber decreases at high frequencies, high absorption performance with wide band is still found.

### Evaluation on methods of estimating the effective depth of a coiled-up cavity

Reliability of the methods for estimating the effective length of the subchannel of a coiled-up cavity mentioned in subsection "Analytical model of the PCD-MPP absorber with coiled-up cavities" is evaluated by comparing with the absorption coefficients between the numerical simulation and analytical model. Specifically, the absorption coefficient of an MPP absorber with a single coiled-up cavity is first evaluated by numerical simulation with the finite element method (FEM) as a benchmark, where the FEM modeling is based on our published work^21^. The proposed analytical model in subsection "Analytical model of the PCD-MPP absorber with coiled-up cavities" is then utilized to calculate theoretical results after obtaining the effective depth of each subchannel.

#### The coiled-up cavity with a coiled-up angle of 180°

**Fig. 4:**
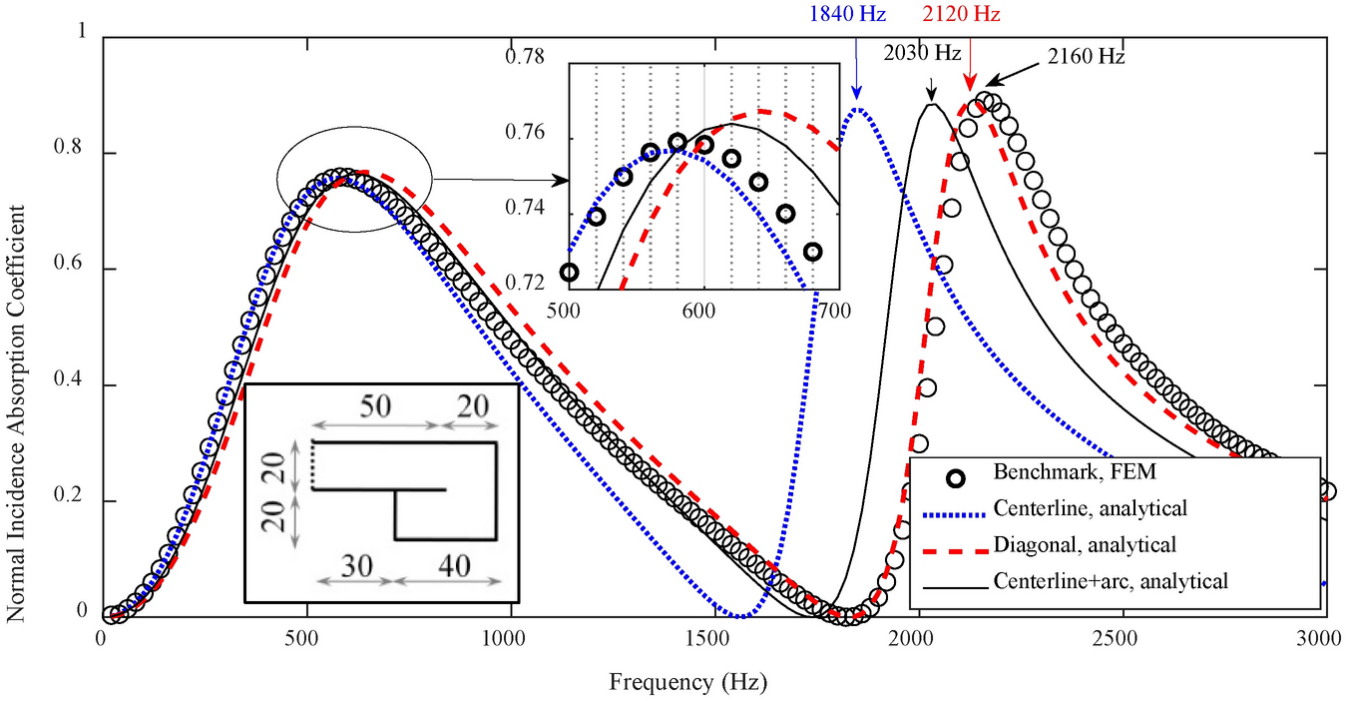
Evaluation on different methods of estimating the effective length of a coiled-up cavity with a coiled-up angle of 180° by comparing the normal incidence absorption coefficients of a single layer MPP absorber. The unit for MPP absorber diagrams in the figure is of millimeter, and the perforation parameters are *d* = *t* = 0.4 mm, *σ* = 1.8%.

**Fig. 5:**
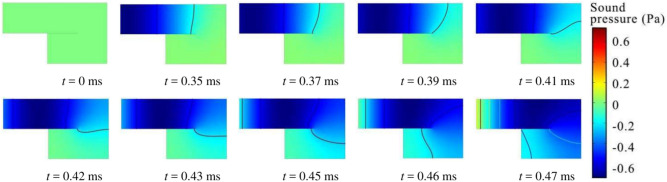
Sound pressure field at 2160 Hz inside the coiled-up cavity at different time by transient simulation, where the colored lines show the wavefront of the sound propagation.

As shown in Fig. 4, evaluation on methods of estimating the effective depth of a coiled-up cavity with the coiled-up angle of 180° is first conducted. Numerical results show that, the predicted result of the absorption coefficient by using the diagonal line as the effective depth of the coiled-up cavity agrees well with the FEM simulation result, with the deviation of resonance frequency lower than 40 Hz. But the result by using the centerline or the combination of centerline and quarter arc shows a poor agreement, with the deviation of the second-order resonance frequency up to 320 Hz. Figure 5 further illustrates the sound pressure field at the second-order resonance frequency (2160 Hz) inside the coiled-up cavity at different time by transient simulations. It can be seen that, the plane wave propagates along the axis of the first subchannel within the period of 0 ~ 0.35 ms. It turns at the inlet of the coiled-up area, and an angle occurs between the wavefront and surface MPP within the period of 0.37 ~ 0.45 ms. The angle decreases when the plane wave propagates into the second subchannel.

**Fig. 6:**
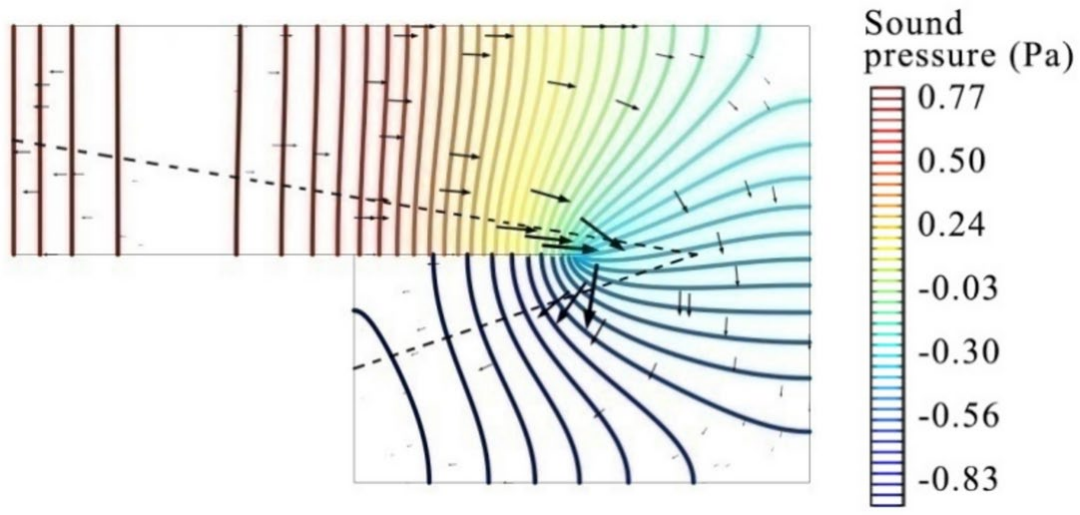
Steady sound pressure field and sound acceleration at 2160 Hz inside the coiled-up cavity by steady-state simulation. Sound pressure values equal on each colored line; black thick arrows represent the sound acceleration amplitude and direction; the black dotted line represents the effective length by using the length of diagonal lines of subchannels.

To better understand the sound propagation path inside the coiled-up cavity, Fig. 6 shows the steady sound pressure field and sound acceleration distribution at 2160 Hz by steady-state simulation, where each colored line represents the wavefront with the equal sound pressure on it. Thick black arrows represent the magnitude and direction of sound acceleration, and the black dotted line denotes the effective depth of the coiled-up cavity by using the diagonal line of each subchannel. It can be observed that, the dotted line representing the effective depth is perpendicular to the wavefronts in the second subchannel and at the end of the first subchannel. The distribution area of sound acceleration with large values (thicker black arrow) matches with the location of the dotted line, and the sound acceleration direction is parallel to the dotted line. We may conclude from the above analysis that, the diagonal line of each subchannel accurately represents the actual propagation path of the plane wave in the coiled-up cavity with a coiled-up angle of 180° than other two methods, and it exactly explain the good agreement of the absorption coefficient between the simulation and analytical results by using this method.

#### The coiled-up cavity with a coiled-up angle of 90°

**Fig. 7:**
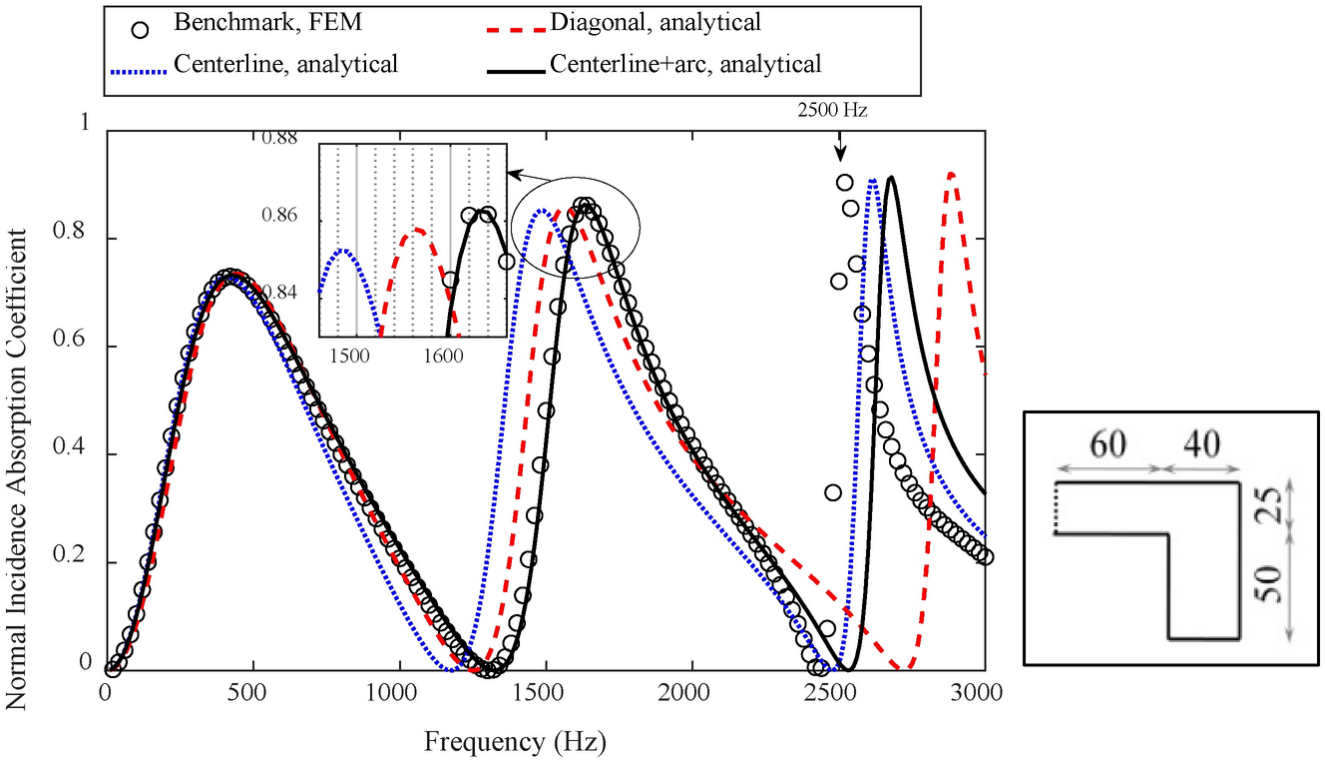
Evaluation on different methods of estimating the effective length of a coiled-up cavity with a coiled-up angle of 90° by comparing the normal incidence absorption coefficients of a single layer MPP absorber, where the unit for MPP absorber diagrams and perforation parameters are equal to that in Fig. 4.

**Fig. 8:**
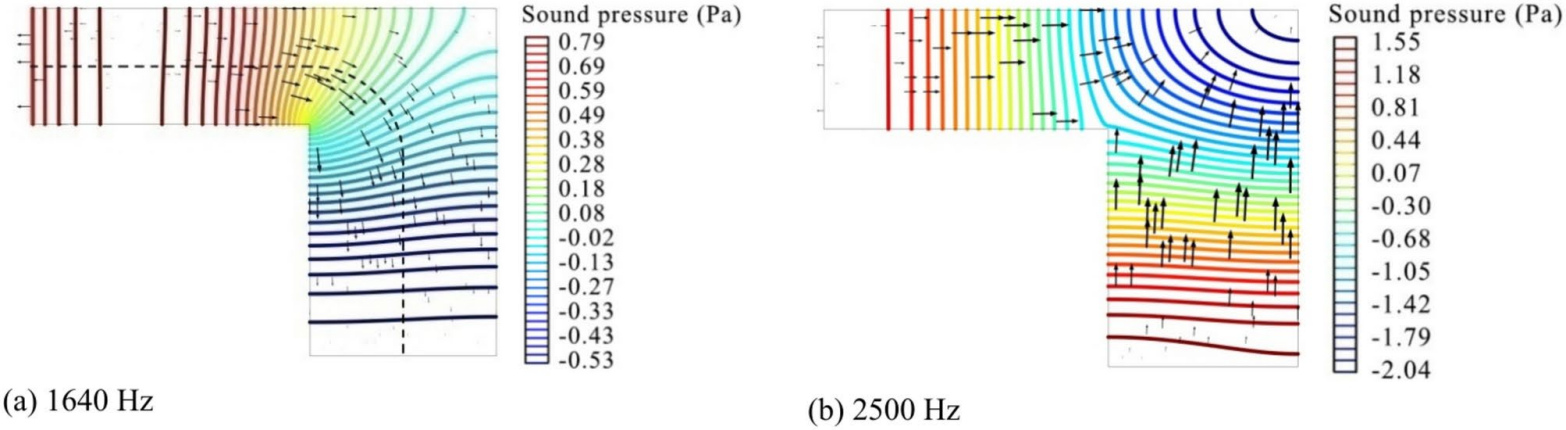
Steady sound pressure field and sound acceleration at (**a**) 1640 Hz and (**b**) 2500 Hz inside the coiled-up cavity by steady-state simulation, the same definitions of colored lines and black thick arrows as described in Fig. 6, and the black dotted line represents the effective length by using the combination of centerlines of subchannels and quarter arc.

When the coiled-up angle of a coiled-up cavity reduces to 90°, as shown in Fig. 7, the analytical result by using the method of combining the centerline with quarter arc agrees perfectly with the FEM simulation result at the first two resonance frequencies, but the result obtained by using the centerline or diagonal line shows a poor agreement at the second-order resonance frequency, with a shift rate of 9.8% and 4.9%, respectively. To better understand the actual sound propagation path inside the coiled-up cavity, Fig. 8a shows the steady sound pressure field and sound acceleration at the second resonance frequency, 1640 Hz, by steady-state simulation, where the black dotted line denotes the effective length by using the combination of centerlines and quarter arc. The plane wave propagates along the axis at the inlet of the first subchannel, and turns in a circular arc at the first subchannel outlet and in the coiled-up area, and finally propagates parallel to the axis of the second subchannel. It can be observed that the dotted line representing the effective length is perpendicular to the wavefront throughout the coiled-up cavity, and parallel to the direction of sound acceleration.

It is noted that the analytical result obtained by using the method of combining the centerline with quarter arc shows a higher deviation at the third resonance frequency, 2500 Hz, than that by using the centerline. Figure 8b thus graphically explains the deviation by showing the steady sound pressure field and sound acceleration by steady-state simulation. Higher order mode waves in a curved shape are clearly observed in the coiled-up area of the coiled-up cavity at the frequency of 2500 Hz, which is quite different from the wavefront in the subchannels. Instead of propagating in an arc path in the coiled-up area as shown with the dotted line in Fig. 8a, the sound wave is more likely reflected at the corner of the coiled-up area and propagating toward the opposite sides in a straight path. Under this situation, a bigger deviation is thus occurred at 2500 Hz in Fig. 7 by using the method of combining the centerline with quarter arc than that by using the centerline. Therefore, under the condition of plane wave propagation, the method of combining the centerline with quarter arc for estimating the effective length of the coiled-up cavity with a coiled-up angle of 90° is more suitable than other methods.

### Reliability of the analytical model of the PCD-MPP absorber with coiled-up cavities

**Fig. 9:**
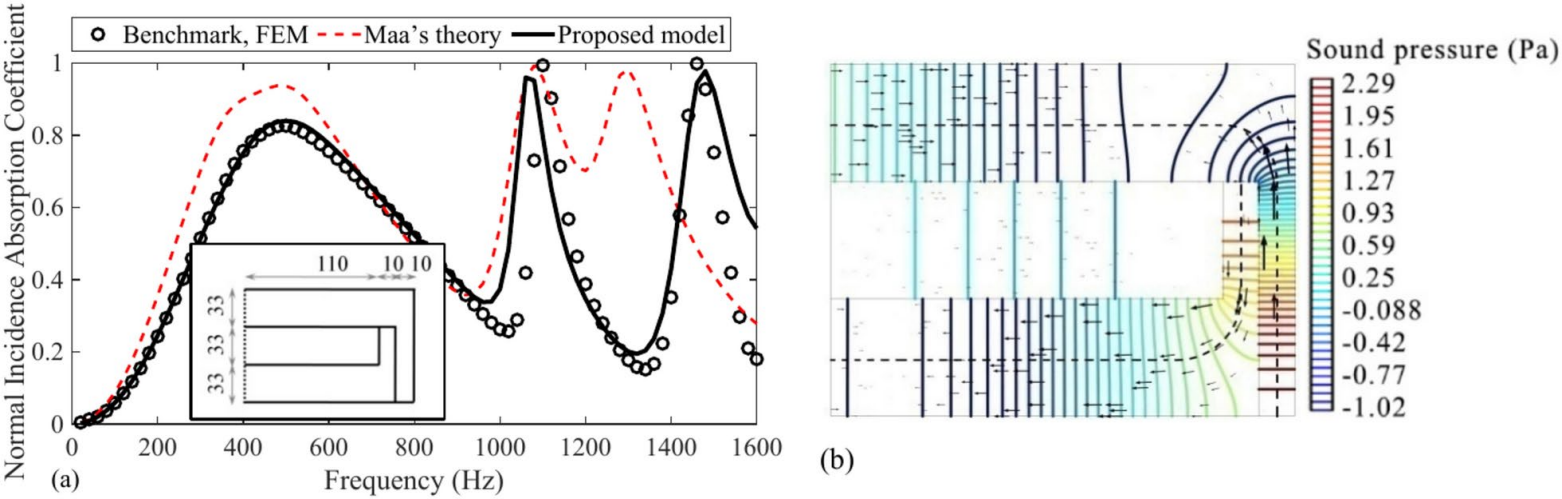
(**a**) Accuracy of the proposed analytical model on evaluating the absorption coefficient of a PCD-MPP absorber with coiled-up cavities. The unit for the PCD-MPP absorber diagram in the figure is of millimeter, and the perforation parameters are *d* = *t* = 0.4 mm, *σ* = 1.8%; (**b**) Steady sound pressure field and sound acceleration at 1080 Hz inside the PCD-MPP absorber with coiled-up cavities by steady-state simulation, the same definitions of colored lines, black thick arrows and black dotted line as described in Fig. 7b.

To investigate the reliability of the analytical model proposed in subsection "Analytical model of the PCD-MPP absorber with coiled-up cavities", the absorption coefficients of a PCD-MPP absorber with coiled-up cavities, obtained by the proposed analytical model, Maa’s theory^33^ and FEM simulation, are presented in Fig. 9, where the effective depth of each subchannel is modeled with the combination of centerlines and quarter arcs. It is revealed that, the absorption coefficient evaluated by the proposed analytical model agrees well with the simulation result, with a shift rate lower than 1.8% of the resonance frequency. However, dramatic errors in absorption coefficient and resonance frequencies are observed by Maa’s theory. As shown in Fig. 9b, the dotted line representing the path of effective length is perpendicular to the wavefront throughout the two coiled-up cavities with coiled-up angles of 90°, and is parallel to the direction of sound acceleration. The proposed analytical model based on the impedance transfer method in this work is thus more reliable and applicable for predicting the absorption coefficient of the PCD-MPP absorber with coiled-up cavities than Maa’s theory.

### Optimization for a PCD-MPP absorber with coiled-up cavities

**Fig. 10:**
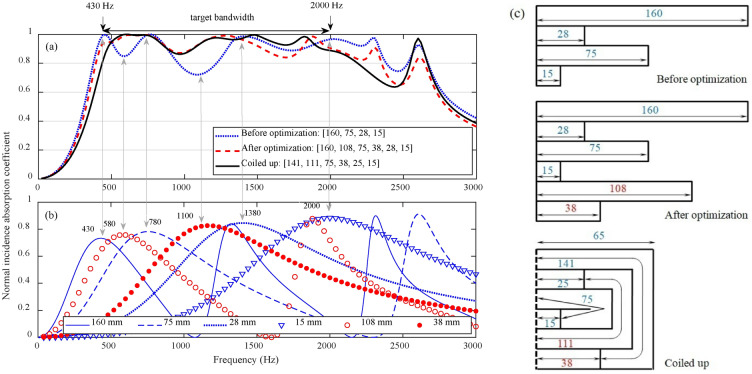
(**a**) Absorption coefficients of a PCD-MPP absorber in different design stages, and (**b**) absorption coefficients of the corresponding single layer MPP absorbers with different cavity depths, where the perforation parameters are: *d* = *t* = 0.4 mm, *σ* = 1.8%; (**c**) schematics of the PCD-MPP absorbers in different design stages.

Based on the theories mentioned in section "Theory", an optimized PCD-MPP absorber with coiled-up cavities upon the given target bandwidth is designed. As shown in Fig. 10, the target bandwidth is assumed within the frequency range of 430–2000 Hz. The first design value of *f*_*α,max,des*_ is thus 430 Hz, and the corresponding upper frequency of the half-absorption bandwith is 780 Hz, which is set as the second design value of *f*_*α,max,des*_. This calculation is repeated until the last design value of *f*_*α,max,des*_ exceeds the upper frequency of the target bandwidth. Hence, four design values of *f*_*α,max,des*_, 430 Hz, 780 Hz, 1380 Hz and 2000 Hz, are preliminarily determined, and a PCD-MPP absorber before optimization is thus obtained with four straight cavities of 160 mm, 75 mm, 28 mm and 15 mm in depth. The corresponding absorption coefficient is shown in Fig. 10, where two valley points situated at 580 Hz and 1100 Hz are observed. Two more design values of *f*_*α,max,des*_ are thus considered, and a new PCD-MPP absorber with six straight cavities is obtained. The absorption coefficient after optimization is larger than 0.8 within the frequency range of 430–2000 Hz. An optimized absorber is finally obtained by coiling up the straight cavities of 160 mm, 28 mm, 108 mm in depth, whose absorption coefficient is larger than 0.8 within the target bandwidth.

**Fig. 11:**
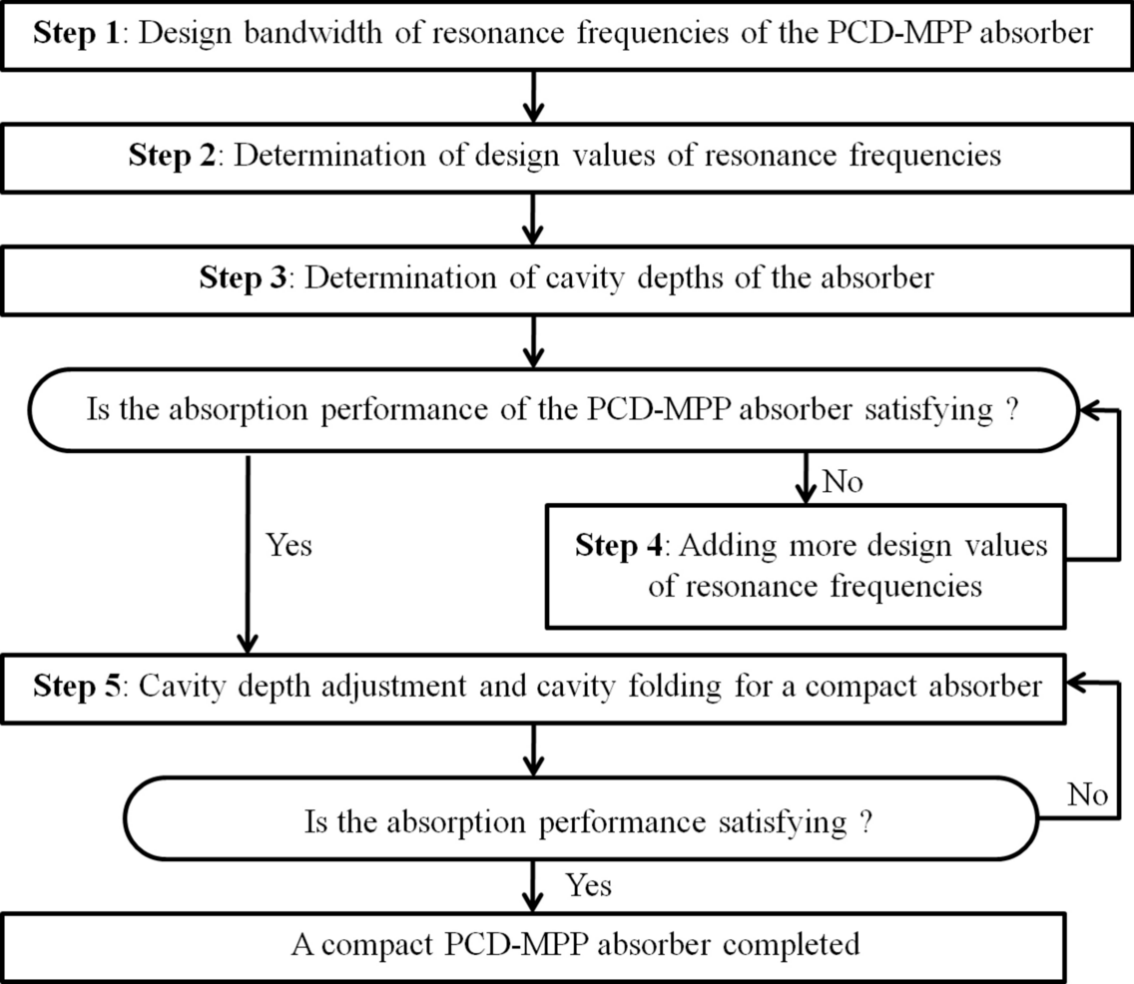
A framework of the design process for a PCD-MPP absorber with coiled-up cavities.

A backing-cavity design method for a PCD-MPP absorber with coiled-up cavities is thus summarized, as shown in Fig. 11. The target bandwidth with high absorption performance is firstly determined based on the noise spectrum. Values of *f*_*α,max,des*_ are preliminarily estimated by using Eq. (15), and then the corresponding cavity depths are calculated using Eq. (16) upon the given perforation parameters, and absorption coefficient of the PCD-MPP absorber is thus obtained. More values of *f*_*α,max,des*_ should be considered if valley points with low absorption coefficients are found within the target bandwidth. To obtain an absorber with a rectangular shape, straight cavities are coiled up and the effective depth is obtained based on the theories in subsection "Analytical model of the PCD-MPP absorber with coiled-up cavities". Evaluations on the absorption performance of the absorber are finally conducted.

## Experiments

**Fig. 12:**
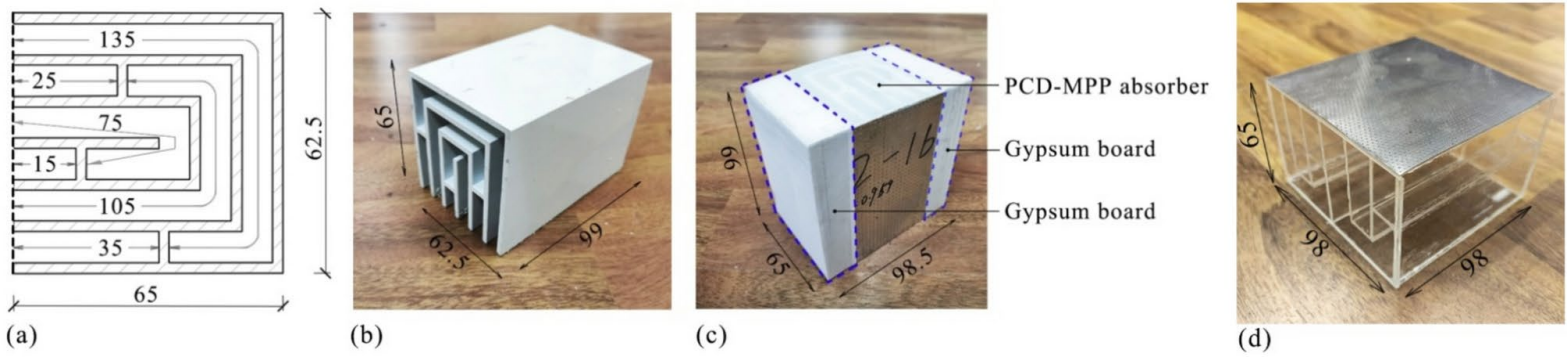
(**a**) The geometry and (**b**) a photograph of the PCD-MPP absorber with coiled-up cavities; (**c**) a photograph of the tested sample; (**d**) a PCD-MPP absorber sample before optimization (units: mm).

The PCD-MPP absorber with coiled-up cavities mentioned in Fig. 10 is produced. As shown in Fig. 12a and b, the PCD, whose size is of 62.5 mm in width, 65 mm in thickness and 99 mm in length, is made of polyvinyl chloride and is partitioned into six 7.5 mm-width curly cavities by 2.5 mm-thickness partition walls. The cavity depth sequence is [135 mm, 25 mm, 75 mm, 15 mm, 105 mm, 35 mm]. A stainless steel MPP, whose size is 62.5 mm × 99 mm, is mounted on the surface of the PCD, and the parameter of the MPP is [*d* = 0.4 mm, *t* = 0.4 mm, *σ* = 1.8%]. Another PCD-MPP absorber sample before optimization, as shown in Fig. 12d, is also fabricated, whose size is of 62.5 mm × 99 mm in cross section, 65 mm in thickness. It has equal perforation parameters, cavity number, cavity width and wall thickness to the sample in Fig. 12a, and the cavity depth sequence is [100 mm, 45 mm, 20 mm, 95 mm, 45 mm, 25 mm].

**Fig. 13:**
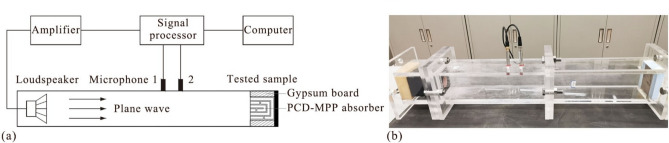
(**a**) A schematic diagram and (**b**) a photograph of the experiment^42^.

Experiments were performed to measure normal incidence sound absorption coefficient of the PCD-MPP absorber in impedance tube^41^. As shown in Fig. 13a, the test rig is designed to be a straight rectangular duct with a cross section of 100 mm × 100 mm, and the wall of the duct is made of 15 mm-thick acrylic to ensure enough sound insulation. The cut-on frequency in this duct is approximately 1700 Hz, and the effective measurement range is from 50 to 1600 Hz. Two 1/2 in. (1.27 cm) microphones Type CHZ-212 + YG-201 were mounted on the tube wall and were flush with the interior surface of the wall to measure the acoustic pressures inside the duct. The distance between the two microphones is 50 mm. One 3.5-inch (88.9 mm) loudspeaker Type HiVi-M3S was mounted on the end of the duct and was driven by the amplifier Type S.M.S.L SA-36A Pro, and the sound signal was produced and processed by the B&K PULSE 3560C system. The absorber was mounted at the other side of the impedance tube, where two gypsum boards (18 mm × 99 mm in cross section and 65 mm in thickness of each) were mounted parallel at each side of the absorber to fill the air gaps between the absorber and duct (see Fig. 13a), since the cross section of the absorber, 62.5 mm × 99 mm, is smaller than that of the impedance tube, and the normal incidence sound absorption coefficient of the absorber was calibrated according to Ref.^42^. The tested sample and a photograph of the experiment are shown in Figs. 12c and 13b, respectively.

**Fig. 14:**
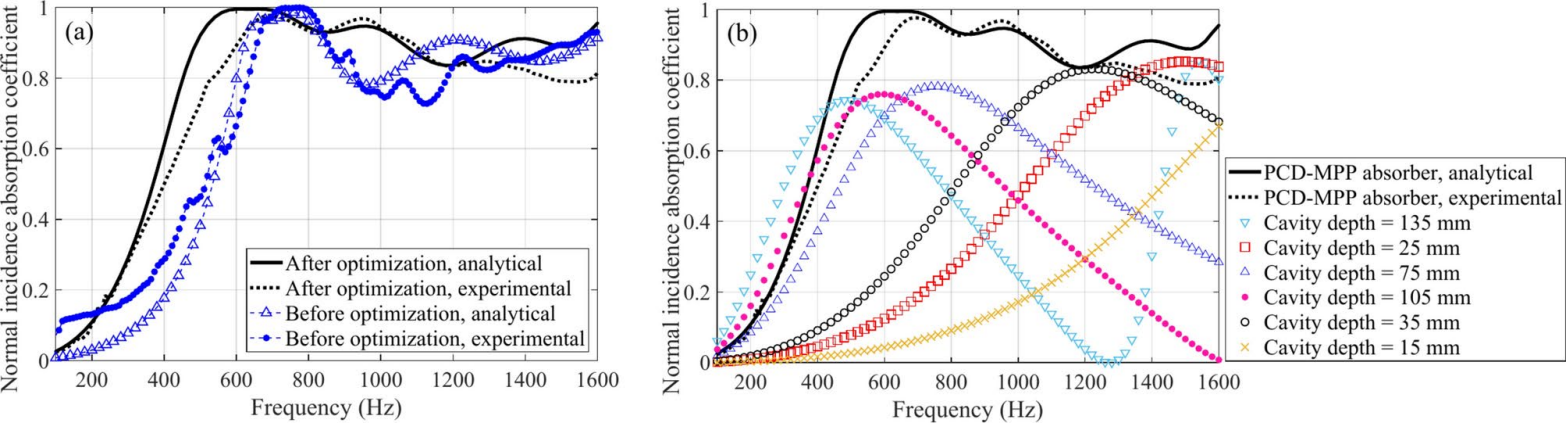
(**a**) Comparisons of the measured and predicted normal incidence sound absorption coefficients of the proposed PCD-MPP absorber after optimization, where the results of the sample before optimization are also presented; (**b**) comparisons of the normal incidence sound absorption coefficients between the PCD-MPP absorber after optimization and single layer MPP absorbers with the corresponding cavity depth.

Figure 14a shows the predicted and measured normal incidence sound absorption coefficients of the PCD-MPP absorber after optimization, where the results of the sample before optimization are also present for comparison. Sound absorption coefficient after optimization is larger than 0.5 within a wide band of 400–1600 Hz, with the maximum and averaged value of 0.98 and 0.91, respectively, while the averaged value within the bandwidth of 400–1600 Hz before optimization is 0.79, indicating the efficiency of the proposed design process in Fig. 11 on absorption performance improvement. Differences between the measured and predicted results are found. Specifically, the first absorption peak by experiment is narrower than that by theoretical prediction, and the absorption coefficients are much lower between 1250 and 1600 Hz. These differences mainly originate from the machining error of the tested sample, as the orifices that produced by laser punching technique may still have ± 10% relative error with the designed requirement, and the acoustic resistance and acoustic mass of the MPP are thus actually different from the designed value. In addition, wall thickness of each cavity is not considered in the analytical model of the PCD-MPP absorber. The error of the measured absorption coefficient is 6.1%, which is evaluated by the formula, ∑*α/*∑*α*_*theo*_^43^, where ∑*α* is the sum of the area between the curves of the theoretical and measured data within the effective band, and ∑*α*_*theo*_ is the total area under the absorption curve by theoretical prediction.

Furthermore, normal incidence absorption coefficients of the corresponding single layer MPP absorbers with the same perforation parameters, predicted by Maa’s method^33^, are also presented in Fig. 14b and compared with the results of the optimized PCD-MPP absorber. It can be seen that if the resonance frequencies of two or more cavities are too close, then the inter-resonator interaction between them will be strong, and only one resonance peak will form from those cavities. For example, the first absorption peak of the PCD-MPP absorber by theoretical prediction is derived from the resonance interaction among the three local resonances of the single MPP absorbers with the cavity depths of 135 mm, 105 mm and 75 mm. While the third peak of the PCD-MPP absorber is derived from the resonance interaction between the local resonances of the other two MPP absorbers and the harmonic of the MPP absorber with 135 mm-depth cavity as well. Therefore, wideband absorption is thus achieved.

## Conclusions

In this paper, design method of PCD-MPP absorbers is studied for sound absorption optimization, particularly on the absorber coiled-up sub-cavities. A prediction model is proposed for estimating the absorber effective sub-cavity depths upon normal incidence absorption coefficient evaluation. FEM simulations and experimental measurements were conducted for validations. Results show that, absorption coefficient of PCD-MPP absorbers by the proposed analytical model agrees well with FEM simulations and experiment measurements. It is also shown that, for the effective depth evaluation of the coiled-up cavities, the diagonal lines of subchannels of coiled-up cavities with a coiled-up angle of 180° can accurately represent the effective depths, while the combination of centerlines of subchannels and quarter arc inside the coiled-up area is more suitable for those with a coiled-up angle of 90°. Optimization investigation shows that, PCD-MPP absorbers can have high absorption performance with the averaged and maximum absorption coefficient of 0.91 and 0.98 within the target bandwidth of 400–1600 Hz, while the absorber thickness can stay below 65 mm. This work can provide valuable guidelines for the effective design of PCD-MPP absorbers with coiled-up cavities for better indoor acoustic quality.

## Data Availability

The datasets generated during the current study are available from the corresponding author on reasonable request.
